# Automatic Analysis of EEGs Using Big Data and Hybrid Deep Learning Architectures

**DOI:** 10.3389/fnhum.2019.00076

**Published:** 2019-03-12

**Authors:** Meysam Golmohammadi, Amir Hossein Harati Nejad Torbati, Silvia Lopez de Diego, Iyad Obeid, Joseph Picone

**Affiliations:** The Neural Engineering Data Consortium, Temple University, Philadelphia, PA, United States

**Keywords:** electroencephalography, EEG, hidden markov models, HMM, deep learning, stochastic denoising autoencoders, SdA, automatic detection

## Abstract

Brain monitoring combined with automatic analysis of EEGs provides a clinical decision support tool that can reduce time to diagnosis and assist clinicians in real-time monitoring applications (e.g., neurological intensive care units). Clinicians have indicated that a sensitivity of 95% with specificity below 5% was the minimum requirement for clinical acceptance. In this study, a high-performance automated EEG analysis system based on principles of machine learning and big data is proposed. This hybrid architecture integrates hidden Markov models (HMMs) for sequential decoding of EEG events with deep learning-based post-processing that incorporates temporal and spatial context. These algorithms are trained and evaluated using the Temple University Hospital EEG, which is the largest publicly available corpus of clinical EEG recordings in the world. This system automatically processes EEG records and classifies three patterns of clinical interest in brain activity that might be useful in diagnosing brain disorders: (1) spike and/or sharp waves, (2) generalized periodic epileptiform discharges, (3) periodic lateralized epileptiform discharges. It also classifies three patterns used to model the background EEG activity: (1) eye movement, (2) artifacts, and (3) background. Our approach delivers a sensitivity above 90% while maintaining a specificity below 5%. We also demonstrate that this system delivers a low false alarm rate, which is critical for any spike detection application.

## Introduction

Electroencephalograms (EEGs) are used in a broad range of health care institutions to monitor and record electrical activity in the brain using electrodes placed on the scalp. EEGs are essential in diagnosis of clinical conditions such as epilepsy, depth of anesthesia, coma, encephalopathy, and brain death (Yamada and Meng, 2017). Manual scanning and interpretation of EEGs is time-consuming since these recordings may last hours or days. It is also an expensive process as it requires highly trained experts. Therefore, high performance automated analysis of EEGs can reduce time to diagnosis and enhance real-time applications by flagging sections of the signal that need further review. Many methods have been developed over the years (Ney et al., 2016) including time-frequency digital signal processing techniques (Osorio et al., 1998; Gotman, 1999), wavelet analysis (Sartoretto and Ermani, 1999), multivariate techniques based on simulated leaky integrate-and-fire neurons (Schindler et al., 2001; Schad et al., 2008), non-linear dynamical analysis of EEG(Stam, 2005), expert systems that attempt to mimic a human observer (Deburchgraeve et al., 2008) and autoregressive spectral analysis of scalp EEG (Khamis et al., 2009). In spite of recent research progress in this field, the transition of current EEG analysis methodologies to the real-life usage in clinical settings like ICUs has been limited, mainly because of unacceptably high false detection rates (Varsavsky and Mareels, 2006; Hopfengärtner et al., 2007).

Machine learning has made tremendous progress over the past three decades due to rapid advances in low-cost highly-parallel computational infrastructure, powerful machine learning algorithms, and, most importantly, big data. Although contemporary approaches for automatic interpretation of EEGs have employed more modern machine learning approaches such as neural networks (Ramgopal, 2014) and support vector machines (Alotaiby et al., 2014), state of the art machine learning algorithms that employ high dimensional models have not previously been utilized in EEG analysis because there has been a lack of large databases that incorporate sufficient real-world variability to adequately train these systems. In fact, what has been lacking in many bioengineering fields including automatic interpretation of EEGs are the big data resources required to support the application of advanced machine learning approaches. A significant big data resource, known as the TUH EEG Corpus (Obeid and Picone, 2016), has recently become available creating a unique opportunity to evaluate high performance deep learning models that require large amounts of training data. This database includes detailed physician reports and patient medical histories, which are critical to the application of deep learning. But, transforming physicians' reports into a deep learning paradigm is proving to be challenging because the mapping of reports to underlying EEG events is non-trivial. Our experiments suggest that a hybrid structure based on hidden Markov models and deep learning can approach clinically acceptable levels of performance.

Spike and seizure detection software is widely used in many countries around the world. Industry leaders such as Persyst (Persyst Development Corporation, 2017) provide a wide variety of tools to automatically detect and classify various EEG events. The limitations of the performance of such systems on tasks such as seizure detection is a widely discussed topic within the clinical and research communities. In fact, in collaboration with IBM, we are hosting a Kaggle-style challenge (see https://www.kaggle.com/) focused on the problem of seizure detection. More details on this challenge will follow in Spring 2019.

## Methods

**Figure 1:**
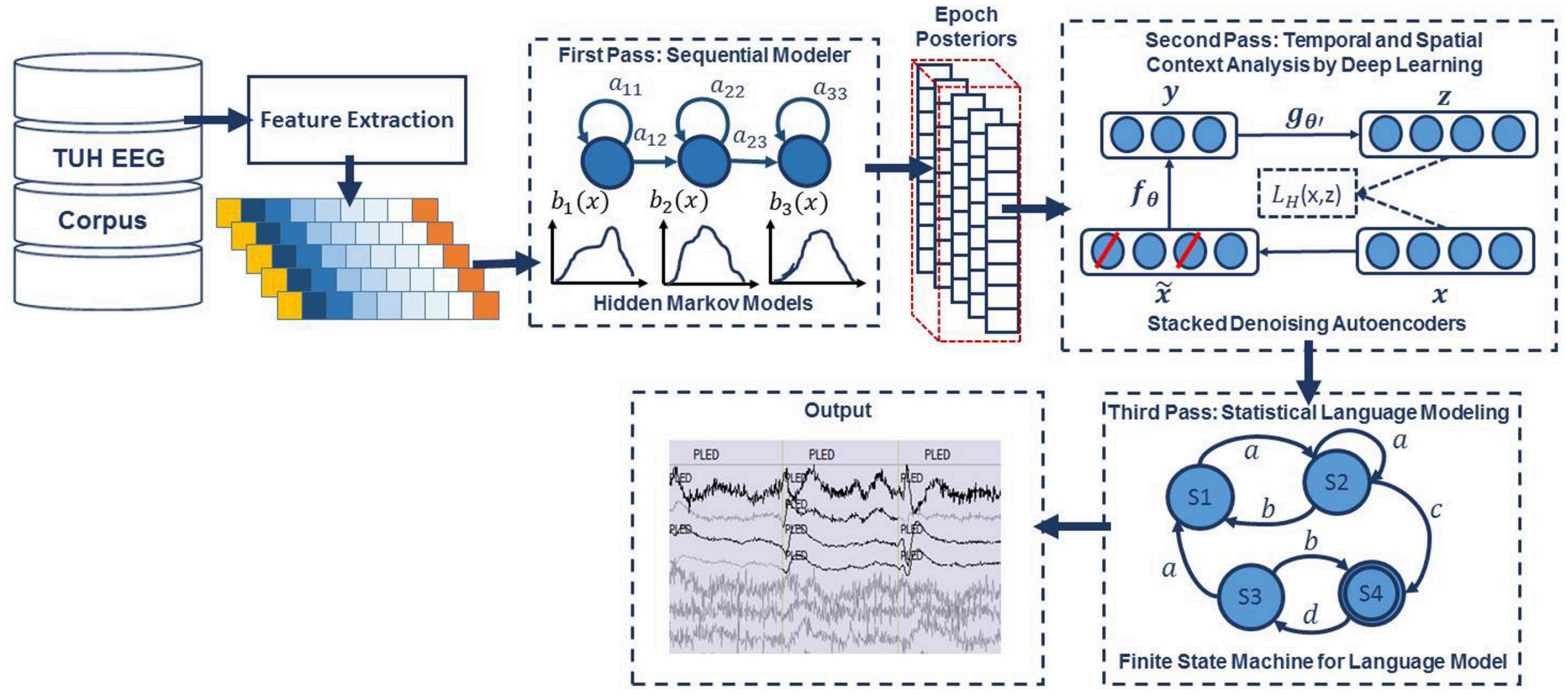
A three-pass architecture for automatic interpretation of EEGs that integrates hidden Markov models for sequential decoding of EEG events with deep learning for decision-making based on temporal and spatial context.

An overview of our proposed system is shown in Figure 1. In order to classify data, N independent feature streams are extracted from the multichannel EEG signal using a standard cepstral coefficient-based feature extraction approach. A sequential modeler analyzes each channel and produces event hypotheses. Three passes of post-processing are performed to produce the final output. In this section, we discuss the various components of this system, including development of the statistical models using a supervised training approach. We begin with a discussion of the data used to train and evaluate the system.

### Data: The TUH EEG Corpus

Our system was developed using the TUH EEG Corpus (TUH-EEG) (Obeid and Picone, 2016), which is the largest publicly available corpus of clinical EEG recordings in the world. The most recent release, v1.1.0, includes data from 2002 to 2015. It contains over 23,000 sessions from over 13,500 patients (over 1.8 years of multichannel signal data in total). This dataset was collected at the Department of Neurology at Temple University Hospital. The data includes sessions taken from outpatient treatments, Intensive Care Units (ICU) and Epilepsy Monitoring Units (EMU), Emergency Rooms (ER) as well as several other locations within the hospital. Since TUH-EEG consists entirely of clinical data, it contains many real-world artifacts (e.g., eye blinking, muscle artifacts, head movements). This makes it an extremely challenging task for machine learning systems and differentiates from most research corpora currently available in this area. Each of the sessions contains at least one EDF file and one physician report. These reports are generated by a board-certified neurologist and are the official hospital record. These reports are comprised of unstructured text that describes the patient, relevant history, medications, and clinical impression. The corpus is publicly available from the Neural Engineering Data Consortium (www.nedcdata.org).

**Figure 2:**
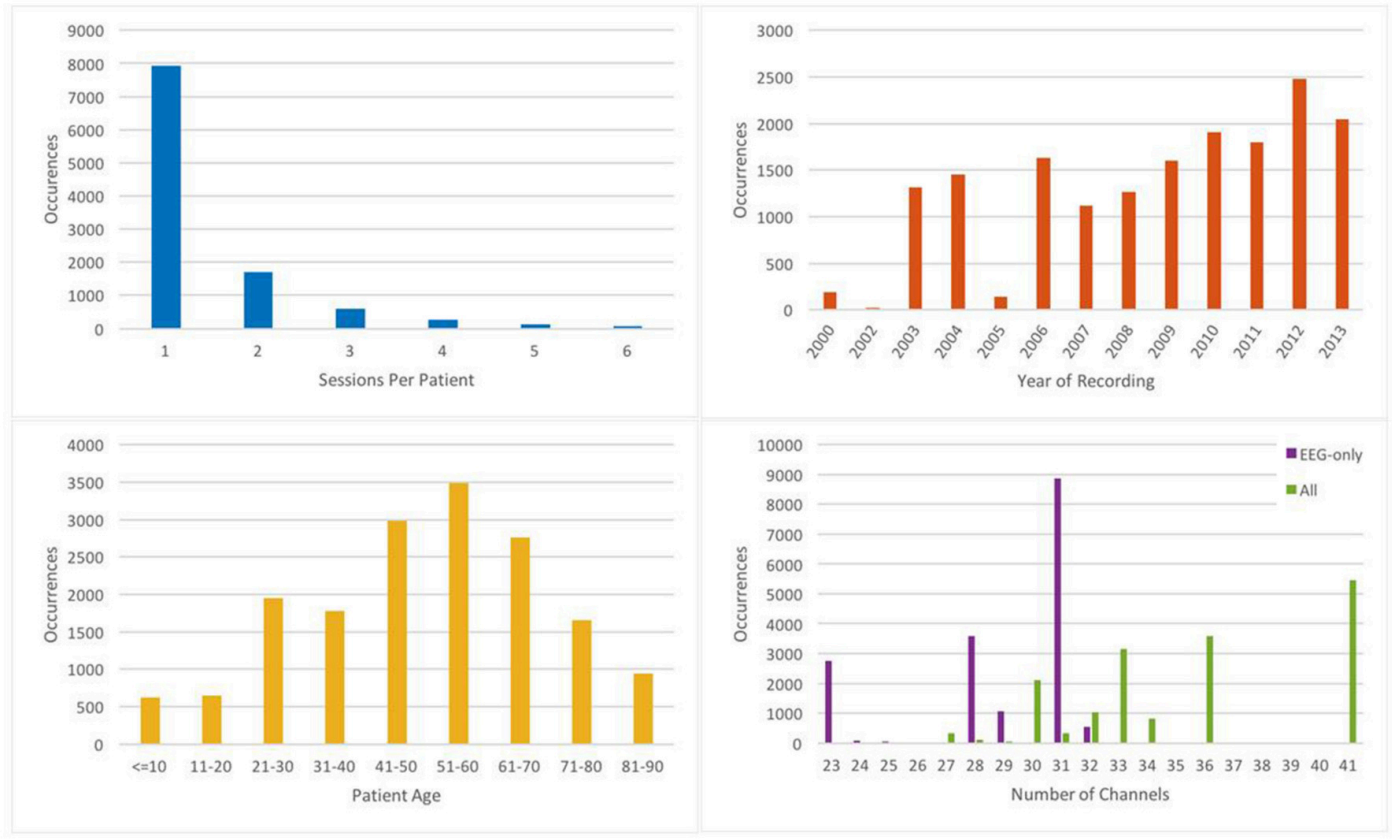
Some relevant statistics demonstrating the variety of data in TUH-EEG.

EEG signals in TUH-EEG were recorded using several generations of Natus Medical Incorporated's Nicolet^TM^ EEG recording technology. The raw signals consist of multichannel recordings in which the number of channels varies between 20 and 128 channels (Harati et al., 2014). A 16-bit A/D converter was used to digitize the data. The sample frequency varies from 250 to 1024 Hz. In our work, we resample all EEGs to a sample frequency of 250 Hz. The Natus system stores the data in a proprietary format that has been exported to EDF with the use of NicVue v5.71.4.2530. The original EEG records are split into multiple EDF files depending on how the session was annotated by the attending technician. Some statistics about the corpus are shown in Figure 2. For our studies, we use the 22 channels associated with a standard 10/20 EEG configuration (American Clinical Neurophysiology Society, 2006).

A portion of TUH-EEG was annotated manually during a study conducted with Temple University Hospital neurologists (Harati et al., 2014). We selected the data based more on the presence of the events of interest described below than the type of EEG since it is difficult to locate examples of spikes. We have analyzed performance as a function of the type/location of the EEG recording for a specific application, seizure detection, using similar technology to that presented in this paper, and not found a significant correlation. The error profiles are similar for EEGs collected in the ICU and EMU from a machine learning perspective.

The annotations we developed comprise six patterns of clinical interest. The first three patterns that might be useful in diagnosing brain disorders are:

1. *spike and/or sharp waves (SPSW):* patterns of EEGs observed during epileptic seizures.
2. *periodic lateralized epileptiform discharges (PLED)*: patterns observed in the context of destructive structural lesions of the cortex. PLED events manifest themselves by presence of a pattern of repetitive periodic, focal, or hemispheric epileptiform discharges like sharp waves, spikes, spike and waves and polyspikes, at intervals of between 0.5 and 3 s.
3. *generalized periodic epileptiform discharges (GPED)*: manifest themselves as periodic short-interval diffuse discharges, periodic long-interval diffuse discharges and suppression-burst patterns. GPEDs are encountered in metabolic encephalopathy and cerebral hypoxia and ischemia. They are similar to PLEDs. In fact, if periodic complexes are limited to a focal brain area they are called as PLEDs, but if periodic complexes are observed over both hemispheres in a symmetric, diffuse and synchronized manner, they are defined as GPEDs.

The other three patterns were used by our machine learning technology to model background noise:

(4) *eye movement (EYEM)*: spike-like signals that occur during patient eye movement.
(5) *artifacts (ARTF)*: recorded electrical activity that is not of cerebral origin including physiologic artifacts generated from sources other than brain. This class also includes extraphysiologic artifacts arising from outside the body such as noise generated from the recording equipment.
(6) *background (BCKG)*: a class used to denote all other data that does not fall in the five classes above. This class usually plays an instrumental role in machine learning systems and needs to include a rich variety of artifacts that are not events of clinical interest.

Note that standard terminology in this field has changed somewhat. PLEDs are now referred to as lateralized periodic discharges (LPDs), GPEDs are now referred to as generalized periodic discharges (GPDs) and spike and sharp waves are referred to as spike and wave (SW) (American Clinical Neurophysiology Society, 2012). However, we will retain the older terminology because this aligns with the way the corpus was annotated and is what was used in our machine learning experiments.

There are over 10 different electrode configurations and over 40 channel configurations represented in the corpus. This poses a serious challenge for machine learning systems since for a system to be practical it must be able to adapt to the specific type of EEG being administered. However, for this initial study, we focused on a subset of the data in which signals were recorded using the Averaged Reference (AR) electrode configuration (Lopez et al., 2016). This data is publicly available at https://www.isip.piconepress.com/projects/tuh_eeg/html/downloads.shtml.

In this paper we focus on the problem of six-way event classification. We have also recently worked on seizure detection using technology that was based on the technology presented here. The work presented here represents our first attempts at doing machine learning on EEG signals and forms the basis for our subsequent work on a wide range of EEG challenges (see https://www.isip.piconepress.com/publications/_index.shtml).

### Data: The TUH-EEG Event Short Set

We collaborated with several neurologists and a team of undergraduate annotators (Shah et al., 2018) to manually label a subset of TUH-EEG for the six different kinds of EEG patterns described in Section Data: The TUH EEG Corpus. This subset, known as the TUH EEG Events Corpus (TUH-EEG-ESS), is available from our project web site: https://www.isip.piconepress.com/projects/tuh_eeg/html/downloads.shtml. The training set is designed to include segments from 359 sessions and the evaluation dataset contains segments from 159 sessions. This data is designed in a way that every patient appears just once in the dataset.

**Figure 3:**
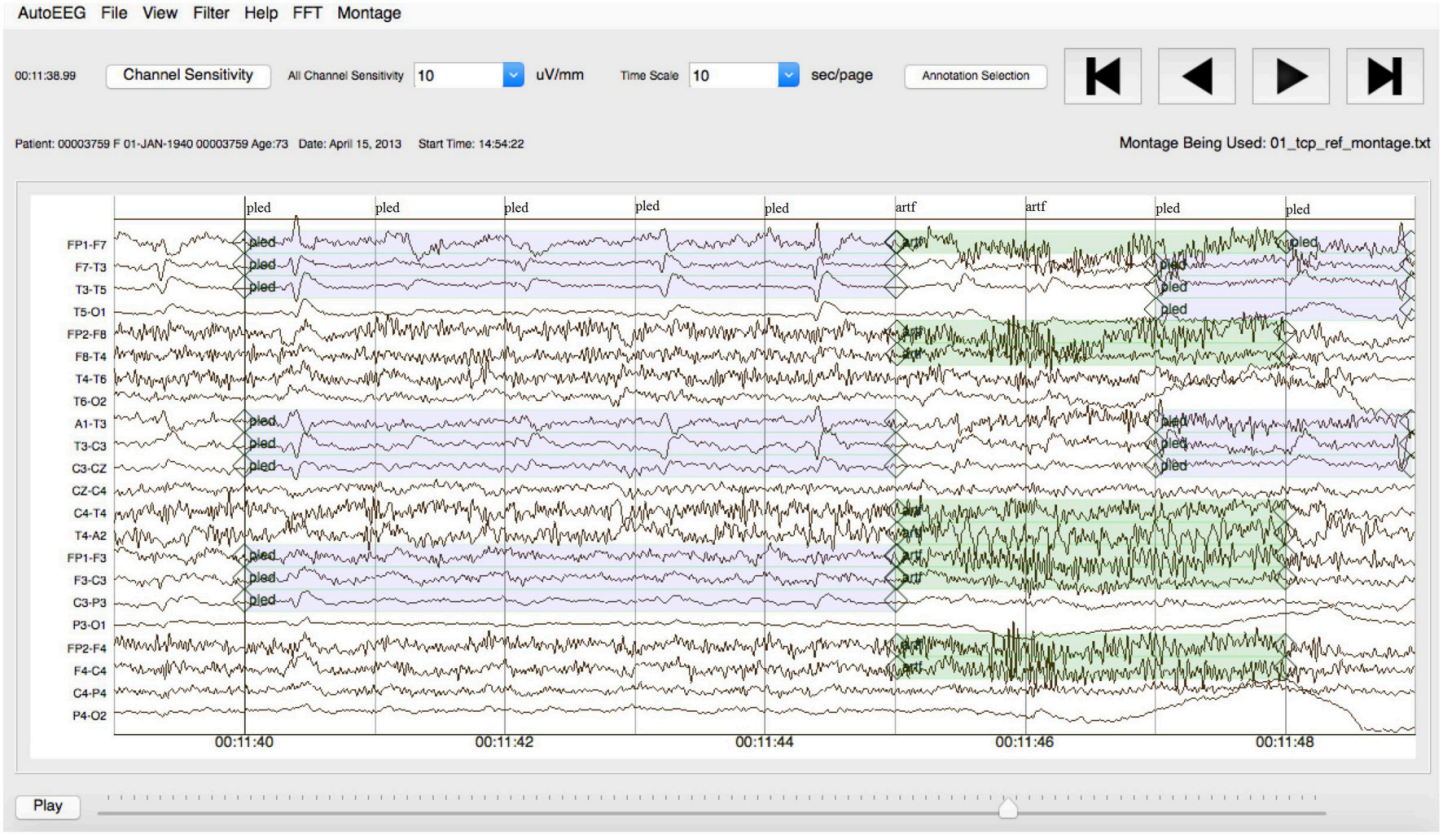
An example demonstrating that the reference data is annotated on a per-channel basis.

Note that the annotations were created on a channel basis—the specific channels on which an event was observed were annotated. This is in contrast to many open source databases that we have observed which only mark events in time and do not annotate the specific channels on which the events occurred. In general, with EEG signals, events such as SPSW do not appear on all channels. The subset of channels on which the event appears is relevant diagnostic information. Our annotations are demonstrated in Figure 3.

A summary of the TU-EEG-ESS dataset is presented in Table 1. The dataset is divided into a training and evaluation set in a way that it includes sufficient number of observations to train machine learning models such as HMMs and evaluate these models on unseen examples from new patients. An overview of the distribution of six types of events for both of training and evaluation set demonstrates that some events occur much less frequently in the actual corpus than other common events. For example, while just <1%of the subset is assigned to SPSW more than 60% is assigned to BCKG. Also notice that 99% of the TU-EEG-ESS data composed of three classes for modeling background which are EYEM, ARTF and BCKG. This distribution of data makes the design of robust classifiers for the detection of non-background classes even more challenging. High performance automatic analysis of EEGs requires dealing with infrequently occurring events since much of the data is uninformative. This is often referred to as an unbalanced data problem, and it is quite common in many biomedical applications. Hence, the evaluation set was designed to contain a reasonable representation of all classes. All of EEGs in this subset were recorded using standard 10–20 system and processed using a TCP montage (Lopez et al., 2016), resulting in 22 channels of signal data per EEG.

**Table 1:** An overview of the distribution of events in the subset of the TUH EEG Corpus used in our experiments.

**Event**	**Train**	**Train % (CDF)**	**Eval**	**Eval % (CDF)**
SPSW	645	0.8% (1%)	567	1.9% (2%)
GPED	6,184	7.4% (8%)	1,998	6.8% (9%)
PLED	11,254	13.4% (22%)	4,677	15.9% (25%)
EYEM	1,170	1.4% (23%)	329	1.1% (26%)
ARTF	11,053	13.2% (36%)	2,204	7.5% (33%)
BCKG	53,726	63.9% (100%)	19,646	66.8% (100%)
Total:	84,032	100.0% (100%)	29,421	100.0% (100%)

### Pre-processing: Feature Extraction

**Figure 4:**
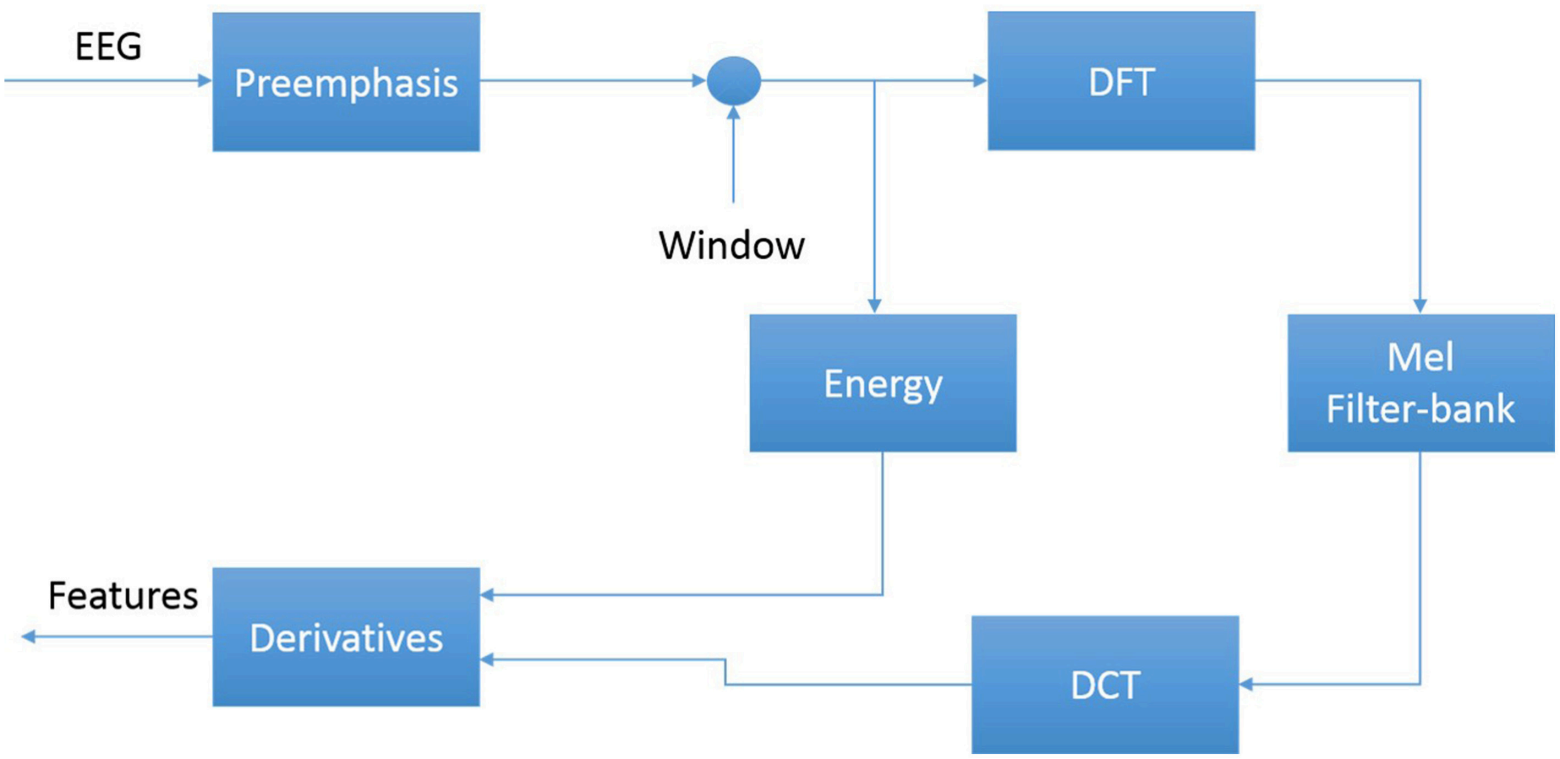
An overview of the feature extraction algorithm.

The first step in EEG processing in Figure 1 consists of converting the signal to a sequence of feature vectors (Picone, 1990). Common EEG feature extraction methods include temporal, spatial and spectral analysis (Mirowski et al., 2009; Thodoroff et al., 2016). A variety of methodologies have been broadly applied for extracting features from EEG signals including wavelet transform, independent component analysis and autoregressive modeling (Jahankhani et al., 2006; Subasi, 2007). In this study, we use a methodology based on mel-frequency cepstral coefficients (MFCC) which have been successfully applied to many signal processing applications including speech recognition (Picone, 1993). In our systems, we use linear frequency cepstral coefficients (LFCCs) since a linear frequency scale provides some slight advantages over the mel scale for EEG signals (Harati et al., 2015). A block diagram summarizing the feature extraction process used in this work for automatic classification of EEG signals is presented in Figure 4. Recent experiments with different types of features (Da Rocha Garrit et al., 2015) or with using sampled data directly (Xiong et al., 2017) have not shown a significant improvement in performance by eliminating the feature extraction process and using sampled data directly.

The first step to derive cepstral coefficients using LFCC feature extraction method is to divide raw EEG signals into shorter frames. The second step is to take a high resolution discreet fast Fourier Transform of each frame. Next, the spectrum is downsampled with a filter bank composed of an array of overlapping bandpass filters. Finally, the cepstral coefficients are derived by computing a discrete cosine transform of the filter bank's output (Picone, 1993). In our experiments, we discarded the zeroth-order cepstral coefficient. Instead of this term we use a frequency domain energy term which is calculated by adding the output of the oversampled filter bank after they are downsampled:

(1) $$E_{f}=log\;(\sum_{k=0}^{N-1}{\;|X(k)|}^{2})$$

In our experiments, we found adding a new feature that is able to model the long-term differentiation in energy can improve the results of spike detection significantly. We call this new feature as differential energy term which can differentiate between transient pulse shape patterns and stationary background noise. To compute differential energy term, we compute the energy of frames inside a window of a channel of EEG. Differential energy equals to maximum energy minus minimum energy over this interval:

(2) $$E_{d}={\text{max}}_{\text{m}}\;(E_{f}\left(m\right))-{\text{min}}_{\text{m}}\;(E_{f}\left(m\right))$$

We have used a window with length of a 0.9 s to calculate differential energy term. Even though this term is a simple feature, our experiments showed that it results in a statistically significant improvement in performance (Harati et al., 2015).

Our experiments have also shown that using derivatives of features based on a regression approach, which is a popular method in speech recognition (Picone, 1993), are effective in the classification of EEG events. We use the following definition for the derivative:

(3) $$d_{t}=\frac{\sum_{n=1}^{N}n\;(c_{t+n}-c_{t-n})}{2\sum_{n=1}^{N}n^{2}}$$

Equation (3) is applied to the cepstral coefficients, *c*_*t*_, to compute the first derivatives, referred to as delta coefficients. Equation (3) is then reapplied to the first derivatives to compute the second derivatives, which are referred to as delta-delta coefficients. We use a window with length of 9 (*N* = 9) for the first derivative and a window with length of 3 (*N* = 3) for the second derivative. The introduction of derivatives helps the system discriminate between steady-state behavior, such as that found in a PLED event, and impulsive or non-stationary signals, such as that found in spikes (SPSW) and eye movements (EYEM).

In this work, through experiments designed to optimize feature extraction, we found best performance can be achieved using a feature vector length of 26. This vector includes nine absolute features consisting of seven cepstral coefficients, one frequency-domain energy term, and one differential energy term. Nine deltas are added for these nine absolute features. Eight delta-deltas are added because we exclude the delta-delta term for differential energy (Harati et al., 2015).

### First Pass: Sequential Decoding Using Hidden Markov Models

Hidden Markov Models (HMMs) are one of the most important machine learning models available today for sequential machine learning problems that require both temporal and spectral modeling (Picone, 1990; Juang and Rabiner, 1991). HMMs can be considered as a class of doubly stochastic processes that are able to model discrete state sequences as Markov chains. HMMs have been used broadly in speech recognition where a speech signal can be decomposed into an energy and frequency profile in which particular events in the frequency domain can be used to identify the sound spoken.

The challenge of interpreting and finding patterns in EEG signal data is very similar to that of speech related projects. There is one distinct difference, however. In a typical speech signal, speech comprises about 50% of the signal and speech events occur frequently. In EEG signals, key events such as seizures occur <1% of the time. This disparity in prior probabilities of these events makes training somewhat of a challenge, since there is overwhelming pressure for the system to simply ignore the events of interest.

For automatic analysis of EEGs, we consider EEG signals to bare composed of a chain of encoded messages as a sequence of one or more symbols. We model an EEG as a sequence of one of six symbols: SPSW, PLED, GPED, EYEM, ARTF, and BCKG. We assume that each one of these patterns is represented by a sequence of feature vectors or observations O, defined as:

(4) $$O=o_{1},\;o_{2},\ldots ,\;o_{T}$$

Here o_T_ is the feature vector observed at time t. If we define S_i_ as the ith event in our dictionary of K events, and S as a sequence of events from this dictionary, then the EEG pattern recognition problem can be considered as finding the most probable sequence of events that maximize the posterior probability P(O | S). We train one HMM model for each event in our dictionary using manually annotated data.

**Figure 5:**
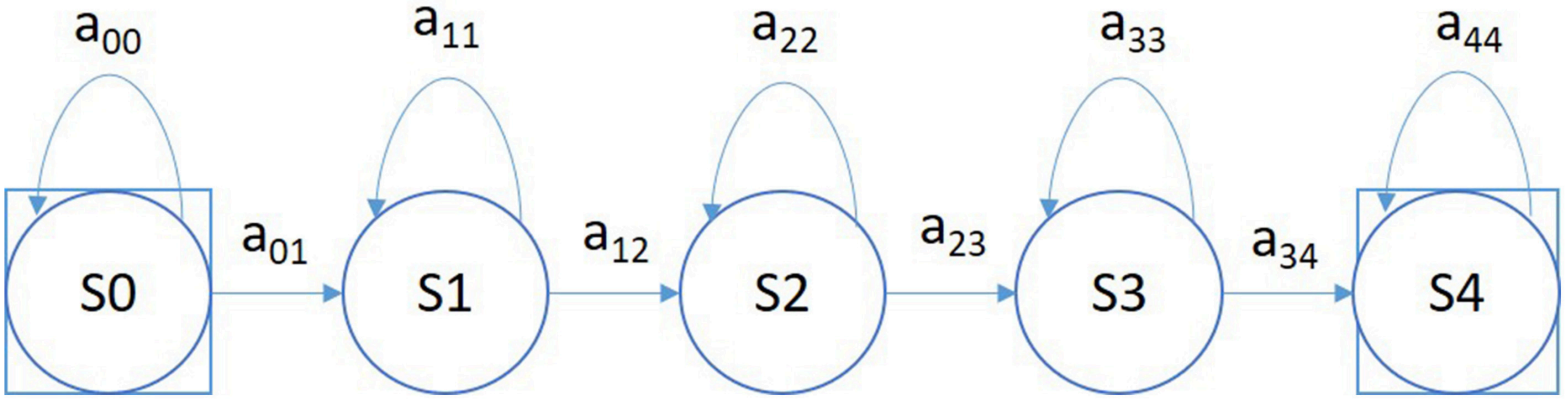
A left-to-right HMM is used for sequential decoding in the first pass of processing.

A simple left-to-right GMM-HMM, illustrated in Figure 5, was used for sequential decoding of EEG signals. A GMM-HMM is characterized by N states where each state consists of an L-component Gaussian mixture model. The transition probability matrix which describes how the states are interconnected consists of a set of probabilities a_ij_ which denotes the probability of a transition from state i to j. Considering α (i, t) as the forward probability where (i = 1, 2,…,N; t = 1, 2, …, T) , β (j, t) as the backward probability where (j = 1, 2, …, N; t = T-1, …,0), and P(O|M) as the probability that model M generates symbol series O, the probability that there will be a transition from state i to state j at time t can be defined as:

(5) $$\gamma_{i}\left(i,j\right)=\frac{\alpha \left(i,t-1\right)a_{ij}b_{ij}(O_{t},\mu_{ij},\Sigma_{ij})\beta \left(j,t\right)}{P(O|M)}$$

The reestimation formulae for the transition probabilities are:

(6) $$a_{ij}=\frac{\sum_{t}\gamma_{i}\left(i,j\right)}{\sum_{t}\sum_{j}\gamma_{i}\left(i,j\right)}$$

We can calculate the output density function using the output vector, O_t_, if it follows an n-dimensional normal distribution as:

(7) $$b_{ij}(o_{t},\mu_{ij},\Sigma_{ij})=\frac{exp\{-{\left(o_{t}-\mu_{ij}\right)}^{t}\sum_{ij}^{-1}(o_{t}-\mu_{ij})/2}{{\left(2\pi \right)}^{n/2}{\left|\Sigma_{ij}\right|}^{1/2}}$$

where μ_ij_ is the mean and Σ_ij_ is the covariance matrix. The mean and covariance for each Gaussian mixture component can be estimated by:

(8) $$\mu_{ij}=\frac{\sum_{t}\gamma_{i}\;(i,j)o_{t}}{\sum_{t}\gamma_{i}\;(i,j)}$$

(9) $$\Sigma_{ij}=\frac{\sum_{t}\gamma_{i}\;(i,j)(o_{t}-\mu_{ij}){\left(o_{t}-\mu_{ij}\right)}^{t}}{\sum_{t}\gamma_{i}\;(i,j)}$$

In the first pass of signal modeling shown in Figure 1, we divide each channel of the EEG signal into epochs. Each epoch is represented by a sequence of frames where each frame is represented by a feature vector. During training, we estimate the parameters of the K models ({a_ij_}, {b_ij_}, { μ_ij_} and { Σ_ij_}) from the training dataset by iterating over all epochs using Equations (5–9). To determine these parameters in an iterative fashion, it is first necessary to initialize them with a carefully chosen value (Picone, 1990). Once this is done, more accurate parameters, in the maximum likelihood sense, can be found by applying the so-called Baum-Welch reestimation algorithm (Picone, 1990). Decoding is typically performed using the Viterbi algorithm (Alphonso and Picone, 2004). Using one HMM model per label, we generate one posterior probability for each model and we select the label that corresponds to the highest probability. Rather than use the best overall output from the HMM system, we let the HMM system output probabilities for each event for each epoch for each channel, and we postprocess these probabilities using a second pass consisting of a deep learning-based system.

### Second Pass: Temporal and Spatial Context Analysis Based on Deep Learning

The goal of the second pass of processing in Figure 1 is to integrate spatial and temporal context to improve decision-making. Therefore, the output of the first pass of processing, which is a vector of six posterior probabilities for every epoch of each channel, is postprocessed by a deep learning system. This system extracts knowledge in a data-driven manner and learn representations of data that involve multiple levels of abstraction (LeCun et al., 2015).

In the second pass of processing, we are using a specific type of deep leaning network known as a Stacked denoising Autoencoder (SdA) (Vincent et al., 2010). SdAs have proven to perform well for applications where we need to emulate human knowledge (Bengio et al., 2007). Since interrater agreement for annotation of seizures tends to be relatively low and somewhat ambiguous, we need a deep learning structure that can deal with noisy inputs. From a structural point of view, SdAs are composed of multiple layers of denoising autoencoders in a way that the input to each layer is the latent representation of the denoising autoencoder found in the layer below. The most important feature of denoising autoencoders that make them appropriate for automatic analysis of EEGs is their ability in reconstructing a repaired input from a corrupted version of it.

Denoising Autoencoders are themselves an extension of a classical autoencoder (Vincent et al., 2008). The input vector to an autoencoder is x ∈ [0, 1]^d^. Then using a deterministic mapping, autoencoder maps the input to a hidden representation y ∈ [0, 1]^d^′ as:

(10) $$y=f_{\theta }\;(x)=s(Wx+b)$$

where *W* is a *d*′ × *d* weight matrix, *b* is a bias vector, *s* is a non-linearity such as sigmoid function and θ = {*W, b*}.

A decoder maps this latent representation y to a reconstruction z of the same shape as x:

(11) $$z=g_{\theta^{\prime }}\;(y)=s\;(W^{\prime }\;y+b^{\prime })$$

It is common to constrain this mapping using a technique by applying a constraint on these equations such as. This particular constraint is known as tied weights. The parameters of this model are optimized to minimize the average reconstruction error using a loss function, *L*, such as reconstruction cross-entropy:

(12) $$\theta^{*},\theta^{\prime *}=\arg\;\min_{\theta ,\;\theta^{\prime }}\frac{1}{n}\underset{i=1}{\sum^{n}}L(x^{\left(i\right)},\;g_{\theta^{\prime }}\;(f_{\theta }\left(x^{\left(i\right)}\right)))$$

**Figure 6:**
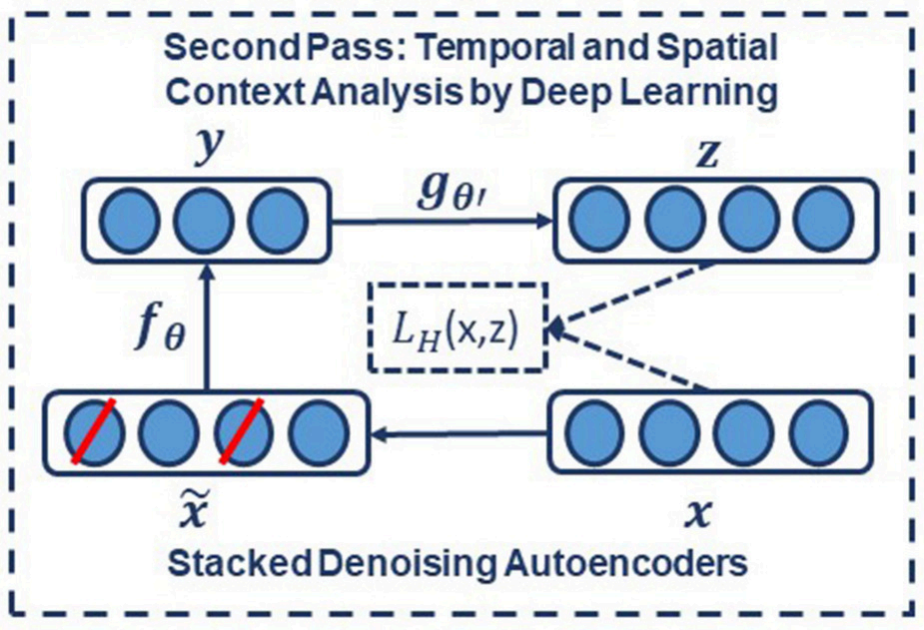
In a stacked denoising autoencoder the input, x, is corrupted to $\tilde{x}$. The autoencoder then maps it to y and attempts to reconstruct x.

To implement a denoising autoencoder, we train an autoencoder on partially corrupted and destroyed input data in a way that it learns to reconstruct a repaired version of the input. To implement this methodology, we use a stochastic mapping function as $\tilde{\text{x}}={\text{q}}_{\text{D}}\left(\tilde{\text{x}}|\text{x}\right)$ for mapping the input x to a partially destroyed version $\tilde{\text{x}}$. We use the corrupted data $\tilde{\text{x}}$ as the input of a typical autoencoder to calculate the latent representation by means of $\text{y}={\text{f}}_{\theta }\left(\tilde{\text{x}}\right)=\text{s}\left(\text{W}\tilde{\text{x}}+\text{b}\right)$. We reconstruct a repaired version of the input using $\text{y}={\text{f}}_{\theta }\left(\tilde{\text{x}}\right)=\text{s}\left(\text{W}\tilde{\text{x}}+\text{b}\right)$. The schematic representation of the process is presented in Figure 6. In the training process, the goal is to find parameters that minimize the loss function which in this case is the average reconstruction error on the training dataset. Note that in these equations, unlike basic autoencoders, reconstruction of z is not a function of x but it is a deterministic function of $\tilde{x}$ and thereby the result of a stochastic mapping of x.

The application of deep learning networks like SdAs generally involves three steps: design, training and implementation. In the design step, the number of inputs and outputs, the number of layers, and the function of nodes are defined. During training, the weights of the nodes are determined through a deep learning process. In the last step, the statistical model is implemented using the fixed parameters of the network determined during training. Pre-processing of the input data is an additional step that is extremely important to various aspects of the deep learning training process.

**Figure 7:**
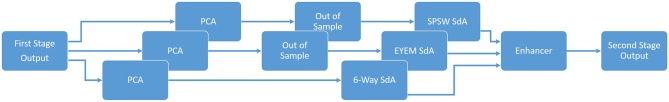
An overview of the second pass of processing.

The block diagram of the second stage of processing is depicted in Figure 7. This stage consists of three parallel SdAs designed to integrate spatial and temporal context to improve decision-making. These SdAs are implemented with varying window sizes to effectively perform a multi-time-scale analysis of the signal and map event labels onto a single composite epoch label vector. A first SdA, referred to as an SPSW-SdA, is responsible for mapping labels into one of two classes: epileptiform and non-epileptiform. A second SdA, EYEM-SdA, maps labels onto the background (BCKG) and eye movement (EYEM) classes. A third SdA, 6W-SdA, maps labels to any one of the six possible classes. The first two SdAs use a relatively short window context because SPSW and EYEM are localized events and can only be detected when we have adequate temporal resolution.

Training of these three SdA networks is done in two steps: pre-training and fine-tuning. SdAs are deep learning networks that are composed of multiple layers of denoising autoencoders. Pre-training is an unsupervised approach that minimizes the reconstruction error. During pre-training, we train each layer of the SdA separately using an unsupervised approach in which we train the first level of a denoising autoencoder to minimize the error in reconstructing of its input. Next, using the output code of the first layer, we train the second layer denoising autoencoder to learn a second level encoding function. This process is repeated for all layers.

Following completion of pre-training, we perform fine-tuning using a supervised training procedure. In fine-tuning the goal is to minimize a loss function that represents the classification error. First, we compose a network with just the encoding parts of each denoising auto-encoder and then we add a logistic regression layer as the last layer of a SdA deep learning network. We initialize this network using weights that we obtained during pre-training and train the entire network to minimize the prediction error (Hinton et al., 2006; Bengio et al., 2007).

As shown in Figure 7, we also preprocess the data using a global principal components analysis (PCA) to reduce dimensionality before application of these SdAs (van der Maaten et al., 2009). PCA is applied to each individual epoch by concatenating each channel output into a supervector and then reducing its dimensionality. For rare and localized events (e.g., SPSW and EYEM), we use an out-of-sample technique to increase the number of training samples (van der Maaten et al., 2009).

Finally, using a block called an enhancer (Vincent et al., 2010), the outputs of these three SdAs are then combined to obtain the final decision. To add the three outputs together, we initialize our final probability output with the output of the 6-way classifier. For each epoch, if the other two classifiers detect epileptiform or eye movement and the 6-way classifier was not in agreement with this, we update the output probability based on the output of 2-way classifiers. The overall result of the second stage is a probability vector of dimension six containing a likelihood that each label could have occurred in the epoch. It should also be noted that the outputs of these SdAs are a probability vector. A soft decision paradigm is used because this output will be smoothed in the third stage of processing.

### Third Pass: Statistical Language Modeling

Neurologists generally impose certain restrictions on events when interpreting an EEG. For example, PLEDs and GPEDs don't happen in the same session. None of the previous stages of processing address this problem. Even the output of the second stage accounts mostly for channel context and is not extremely effective at modeling long-term temporal context. The third pass of processing addresses this issue and improves the overall detection performance by using a finite state machine based on a statistical language model. In general, for problems such as EEG event detection in which infrequently occurring events play a significant role, post-processing based on domain knowledge tends to provide large gains in performance. Automatic this using deep learning is not trivial.

As is shown in Figure 1, the third stage of post-processing is designed to impose some contextual restrictions on the output of the second stage. These contextual relationships involve long-term behavior of the signal and are learned in a data-driven fashion. This approach is also borrowed from speech recognition where a probabilistic grammar is used that combines the left and right contexts with the labels (Levinson, 2005). This is done using a finite state machine that imposes specific syntactic constraints.

In this study, a bigram probabilistic language model that provides the probability of transiting from one type of epoch to another (e.g., PLED to PLED) is prepared using the training dataset and also in consultation with neurologists in Temple Hospital University. The bigram probabilities for each of the six classes are shown in Table 2, which models all possible transitions from one label to the next. The remaining columns alternate between the class label being transitioned to and its associated probability. The probabilities in this table are optimized on a training database that is a subset of TUH-EEG. For example, since PLEDs are long-term events, the probability of transitioning from one PLED to the next is high ~0.9. However, since spikes that occur in groups are PLEDs or GPEDs, and not SPSWs, the probability of transitioning from a PLED to SPSW is 0.0. Therefore, these transition probabilities emulate the contextual knowledge used by neurologists.

**Table 2:** A bigram probabilistic language model for the third pass of processing which models all possible transitions from one of the six classes to the next.

**i**	**j**	**P(i, j)**	**j**	**P(i, j)**	**j**	**P(i, j)**	**j**	**P(i, j)**	**j**	**P(i, j)**	**j**	**P(i, j)**
SPSW	SPSW	0.40	PLED	0.00	GPED	0.00	EYEM	0.10	ARTF	0.20	BCKG	0.30
PLED	SPSW	0.00	PLED	0.90	GPED	0.00	EYEM	0.00	ARTF	0.05	BCKG	0.05
GPED	SPSW	0.00	PLED	0.00	GPED	0.60	EYEM	0.00	ARTF	0.20	BCKG	0.20
EYEM	SPSW	0.10	PLED	0.00	GPED	0.00	EYEM	0.40	ARTF	0.10	BCKG	0.40
ARTF	SPSW	0.23	PLED	0.05	GPED	0.05	EYEM	0.23	ARTF	0.23	BCKG	0.23
BCKG	SPSW	0.33	PLED	0.05	GPED	0.05	EYEM	0.23	ARTF	0.13	BCKG	0.23

After compiling the probability table, a long window is centered on each epoch and the posterior probability vector for that epoch is updated by considering left and right context as a prior (essentially predicting the current epoch from its left and right context). A Bayesian framework is used to update the probabilities of this grammar for a single iteration of the algorithm:

(13) $$P_{gprior}=\frac{\sum_{i=1}^{L}P_{i}+\epsilon_{prior}M}{L+M}$$

(14) $$RPP(k)=\frac{\beta_{R}\sum_{i=1}^{N}\exp(-i\lambda )\;P_{k+i}+\alpha P_{gprior}}{1+\alpha }$$

(15) $$LPP(k)=\frac{\beta_{L}\sum_{i=1}^{N}\exp\;(-i\lambda )\;P_{k-i}+\alpha P_{gprior}}{1+\alpha }$$

(16) $$P_{C_{k}|LR}=\beta_{C}P_{C_{k}}{\left(\sum_{i=1}^{k}\sum_{j=1}^{k}LPP\;(i)\;RPP(j)Prob\;(i,k)Prob(k,\;j)\right)}^{\frac{\gamma }{n}}$$

In these equations, k = 1, 2… K where K is the total number of classes (in this study K = 6), L is number of epochs in a file, ϵ_prior_ is the prior probability for an epoch (a vector of length K) and M is the weight. LPP and RPP are left and right context probabilities, respectively. λ is the decaying weight for window, α is the weight associated with P_gprior_ and β_R_ and β_L_ are normalization factors. P_C_k__ is the prior probability, P_C_k_|LR_ is the posterior probability of epoch C for class k given the left and right contexts, y is the grammar weight, n is the iteration number (starting from 1) and β_C_ is the normalization factor. Prob(i, j) is a representation of the probability table shown in Table 2. The algorithm iterates until the label assignments, which are decoded based on a probability vector, converge. The output of this stage is the final output and what was used in the evaluations described in Section Results.

## Results

In this section, we present results on a series of experiments designed to optimize and evaluate each stage of processing.

### Pre-processing: Feature Extraction

Features from each epoch are identified using a feature extraction technique described in Section Data: The TUH-EEG Event Short Set. Neurologists review EEGs in 10 s windows. Pattern recognition systems often subdivide the signal into small segments during which the signal can be considered quasi-stationary. HMM systems need further subdivision so that there are enough observations to allow the system to develop a strong sense of the correct choice. A simple set of preliminary experiments determined that a reasonable tradeoff between computational complexity and performance was to split the 10 s window into 1 s epochs, and to further subdivide these into 0.1 s frames. Hence, features were computed every 0.1 s using a 0.2 s overlapping analysis window. The output of the feature extraction system is 22 channels of data, where in each channel, a feature vector of dimension 26 corresponds to every 0.1 s. These parameters were optimized experimentally in a previous study (Harati et al., 2015).

### First Pass: Sequential Decoding Using Hidden Markov Models

A 6-way classification experiment was conducted using the models described in Figure 5. Each state uses 8 Gaussian mixture components and a diagonal covariance assumption (drawing on our experience with speech recognition systems and balancing dimensionality of the models with the size of the training data). Models were trained using all events on all channels resulting in what we refer to as channel independent models. Channel dependent models have not proven to provide a boost in performance and add considerable complexity to the system.

The results for the first pass of processing are shown in Table 3, in the first pass section. A more informative performance analysis can be constructed by collapsing the three background classes into one category. We refer to this second evaluation paradigm as a 4-way classification task: SPSW, GPED, PLED and BACKG. The latter class contains an enumeration of the three background classes. The 4-way classification results for the first pass of processing are presented in Table 4, in the first pass section. Finally, in order that we can produce a detection error tradeoff (DET) curve (Martin et al., 1997) we also report a 2-way classification result in which we collapse the data into a target class (TARG) and a background class (BCKG). The 2-way classification results for the first pass of processing are presented in Table 5, in the first pass section. Note that the classification results for all these tables are measured by counting each epoch for each channel as an independent event. We refer to this as forced-choice event-based scoring because every epoch for every channel is assigned a score based on its class label.

**Table 3:** The 6-way classification results for the three passes of processing.

**Pass**	**Event**	**ARTF**	**BCKG**	**EYEM**	**GPED**	**PLED**	**SPSW**
First	ARTF	41.24	45.19	2.18	3.81	2.77	4.81
	BCKG	7.02	71.93	2.59	7.37	2.28	8.81
	EYEM	2.13	0.61	82.37	2.13	8.51	4.26
	GPED	7.46	4.85	2.39	53.32	20.42	11.55
	PLED	0.70	1.85	4.70	17.62	54.80	20.32
	SPSW	4.41	8.29	9.17	33.33	4.59	40.21
Second	ARTF	27.49	61.73	7.28	0.00	1.08	2.43
	BCKG	7.00	82.03	5.79	0.97	0.36	3.86
	EYEM	4.21	16.84	77.89	0.00	0.00	1.05
	GPED	0.60	14.69	0.00	59.96	10.26	14.49
	PLED	1.40	22.65	0.80	13.83	52.30	9.02
	SPSW	7.69	35.90	2.56	28.21	0.00	25.64
Third	ARTF	14.04	72.98	10.18	0.00	0.00	2.81
	BCKG	3.42	81.40	8.93	0.30	0.00	5.95
	EYEM	2.30	17.24	79.31	0.00	0.00	1.15
	GPED	0.30	3.65	0.00	65.05	13.37	17.63
	PLED	0.00	10.76	0.49	9.78	65.28	13.69
	SPSW	10.00	33.33	13.33	10.00	0.00	33.33

**Table 4:** The 4-way classification results for the three passes of processing.

**Pass**	**Event**	**BCKG**	**SPSW**	**GPED**	**PLED**
First	BCKG	82.30	8.35	6.94	2.42
	SPSW	21.87	40.21	33.33	4.59
	GPED	14.71	11.55	53.32	20.42
	PLED	7.26	20.32	17.62	54.80
Second	BCKG	95.60	3.24	0.62	0.54
	SPSW	46.15	25.64	28.21	0.00
	GPED	15.29	14.49	59.96	10.26
	PLED	24.85	9.02	13.83	52.30
Third	BCKG	95.11	4.69	0.19	0.00
	SPSW	56.67	33.33	10.00	0.00
	GPED	3.95	17.63	65.05	13.37
	PLED	11.25	13.69	9.78	65.28

**Table 5:** The 2-way classification results for the three passes of processing.

**Pass**	**Event**	**TARG**	**BCKG**
First	TARG	86.92	13.08
	BCKG	18.20	81.80
Second	TARG	78.94	21.06
	BCKG	4.40	95.60
Third	TARG	90.10	9.90
	BCKG	4.89	95.11

### Second Pass: Temporal and Spatial Context Analysis Based on Deep Learning

The output of the first stage of processing is a vector of six scores, or likelihoods, for each channel at each epoch. Therefore, if we have 22 channels and six classes, we will have a vector of dimension 6 × 22 = 132 scores for each epoch. This 132-dimension epoch vector is computed without considering similar vectors from epochs adjacent in time. Information available from other channels within the same epoch is referred to as “spatial” context since each channel corresponds to a specific electrode location on the skull. Information available from other epochs is referred to as “temporal” context. The goal of this level of processing is to integrate spatial and temporal context to improve decision-making.

To integrate context, the input to the second pass deep learning system is a vector of dimension 6 x 22 x window length, where we aggregate 132-dimension vectors in time. If we consider a 41-second window, then we will have a 5,412-dimension input to the second pass of processing. This input dimensionality is high even though we have a considerable amount of manually labeled training. To deal with this problem we follow a standard approach of using Principal Components Analysis (PCA) (Fukunaga, 1990) before every SdA. The output of PCA is a vector of dimension 13 for SdA detectors that look for SPSW and EYEM and 20 for 6-way SdA classifier.

Further, since we do not have enough SPSW and EYEM events in the training dataset, we must use an out-of-sample technique (van der Maaten et al., 2009) to train SdA. Three consecutive outputs are averaged, so the output is further reduced from 3 × 13 to just 13, using a sliding window approach to averaging. Therefore, the input for SPSW SdA and EYEM SdA decreases to 13 x window length and 20 x window length for 6-way SdA.

We used an open source toolkit, Theano (Bergstra et al., 2010; Bastien et al., 2012), to implement the SdAs. The parameters of the models are optimized to minimize the average reconstruction error using a cross-entropy loss function. In the optimization process, a variant of stochastic gradient descent is used, referred to as minibatches. Minibatch stochastic gradient descent is similar to stochastic gradient descent, but we use more than one training example to calculate each estimate of the gradient. Using this optimization method, we will have less variance in the estimate of the gradient. Additionally, this framework makes better use of the hierarchical memory organization in modern computers.

SPSW SdA uses a window length of 3 which means it has 39 inputs and 2 outputs. It has three hidden layers with corruption levels of 0.3 for each layer. The number of nodes per layer are: first layer = 100, second layer = 100, third layer = 100. The parameters for pre-training are: learning rate = 0.5, number of epochs = 200, batch size = 300. The parameters for fine-tuning are: learning rate = 0.2, number of epochs = 800 and batch size = 100.

EYEM SdA uses a window length of 3 which means it has 39 inputs and 2 outputs. It has three hidden layers with corruption levels of 0.3 for each layer. The number of nodes per layer are: first layer = 100, second layer = 100, third layer = 100. The parameters for pre-training are: learning rate = 0.5, number of epochs = 200, batch size = 300. The parameters for fine-tuning are: learning rate = 0.2, number of epochs = 100 and batch size = 100.

Six-way SdA uses a window length of 41 which means it has 820 inputs and 6 outputs. It has three hidden layers with corruption levels of 0.3 for each layer. The number of nodes per layer are: first layer = 800, second layer = 500, third layer = 300. The parameters for pre-training are: learning rate = 0.5, number of epochs = 150 and batch size = 300. The parameters for fine-tuning are: learning rate = 0.1, number of epochs = 300 and batch size = 100.

The 6-way, 4-way and 2-way classification results for the second stage of processing are presented in Tables 3–5, in the second pass section, respectively. Note that unlike the tables for the first pass of processing, the classification results in each of these tables are measured once per epoch—they are not per-channel results. We refer to these results as epoch-based.

### Third Pass: Statistical Language Modeling

The output of the second stage of processing is a vector of six scores, or likelihoods, per epoch. This serves as the input for the third stage of processing. The optimized parameters for the third pass of processing are: prior probability for an epoch, ϵ_prior_, is 0.1; the weight, M, is 1; the decaying weight, λ, is 0.2; the weight associated with P_gprior_, α, is 0.1; the grammar weight, y, is 1; the number of iterations, n, is 20, and the window length to calculate the left and right prior probabilities is 10.

The 6-way, 4-way and 2-way classification results are presented in Tables 3–5, in the third pass section, respectively. Note that these results are also epoch-based.

## Discussion

The 6-way classification task can be structured into several subtasks. Of course, due to the high probability of the signal being background, the system is heavily biased toward choosing the background model. Therefore, in Table 4 in the first pass section, we see that performance on BACKG is fairly high. Not surprisingly, BCKG is most often confused with SPSW. SPSW events are short in duration and there are many transient events in BCKG that resemble an SPSW event. This is one reason we added ARTF and EYEM models, so that we can reduce the confusions of all classes with the short impulsive SPSW events. As we annotate background data in more detail, and identify more commonly occurring artifacts, we can expand on our ability to model BCKG events explicitly.

GPEDs are, not surprisingly, most often confused with PLED events. Both events have a longer duration than SPSWs and artifacts. From the first pass section of Table 4, we see that performance on these two classes is generally high. The main difference between GPED and PLED is duration, so we designed the post-processing to learn this as a discriminator. For example, in the second pass of processing, we implemented a window duration of 41 s so that the SdA system would be exposed to long-term temporal context. We also designed three separate SdA networks to differentiate between short-term and long-term context. In Table 4 in the second pass section, we see that the performance of GPEDs and PLEDs improves with the second pass of post-processing. More significantly, the confusions between GPEDs and PLEDs also decreased. Note that also in Table 4 in the second pass section, performance of BCKG increased significantly. Confusions with GPEDs and PLEDs dropped dramatically to below 1%.

While performance across the board increased, performance for SPSW dropped by adding the second pass of post-processing. This is a reflection on the imbalance of the data. Less than one percent of data is annotated as SPSWs, while we have ten times more training samples for GPEDs and PLEDs. Note that we used an out-of-sample technique to increase the number of training samples for SPSWs, but even this technique could not solve the problem of a lack of annotated SPSW data. By comparing the first pass results of Tables 3–5 we saw a similar behavior with the EYEM class because there are also fewer EYEM events.

A summary of the results for different stages of processing is shown in Table 6. The overall performance of the multi-pass hybrid HMM/deep learning classification system is promising: more than 90% sensitivity and <5% specificity.

**Table 6:** Specificity and sensitivity for each pass of processing.

**Pass**	**Sensitivity**	**Specificity**
1 (HMM)	86.78	17.70
2 (SdA)	78.93	4.40
3 (SLM)	90.10	4.88

**Figure 8:**
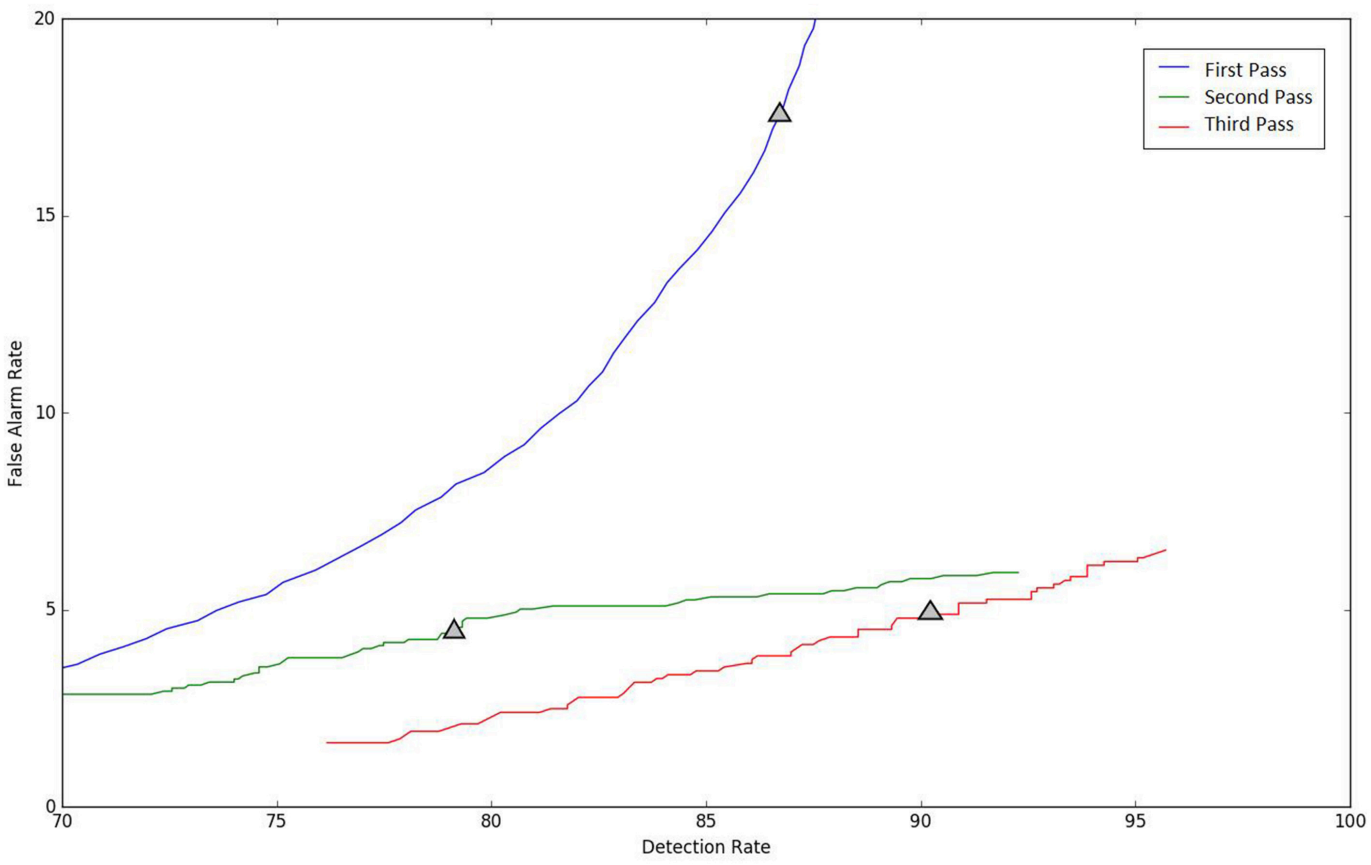
DET curves are shown for each pass of processing. The “zero penalty” operating point is also shown since this was used in Tables 3–5.

Because the false alarm rate in these types of applications varies significantly with sensitivity, it is important to examine performance using a DET curve. A DET curve for the first, second, and third stage of processing is given in Figure 8. Note that the tables previously presented use the unprocessed likelihoods output from the system. They essentially correspond to the point on the DET curve where a penalty of 0 is applied. This operating point is identified on each of the curves in Figure 8. We see that the raw likelihoods of the system correspond to different operating points in the DET curve space. From Figure 8 it is readily apparent that post-processing significantly improves our ability to maintain a low false alarm rate as we increase the detection rate. In virtually all cases, the trends shown in Tables 3–6 hold up for the full range of the DET curve. This study demonstrates that a significant amount of contextual processing is required to achieve a specificity of 5%.

## Conclusion

Virtually all previous R&D efforts involving EEG, including seizure detection, have been conducted on small databases (Akareddy et al., 2014). Often these databases are not good representations of the type of data observed in clinical environments. Transient artifacts, not common in databases collected under research conditions, can significantly degrade performance. Not surprisingly, despite high accuracies presented in the research literature, the performance of commercially available systems has been lacking in clinical settings. There is still great demand for an automated system that achieves a low false alarm rate in clinical applications.

We have presented a three-pass system that can achieve high performance classifying EEG events of clinical relevance. The system uses a combination of HMMs for accurate temporal segmentation and deep learning for high performance classification. In the first pass, the signal is converted to EEG events using an HMM-based system that models the temporal evolution of the signal. In the second pass, three stacked denoising autoencoders (SDAs) with different window durations are used to map event labels onto a single composite epoch label vector. We demonstrated that both temporal and spatial context analysis based on deep learning can improve the performance of sequential decoding using HMMs. In the third pass, a probabilistic grammar is applied that combines left and right context with the current label vector to produce a final decision for an epoch.

Our hybrid HMM/deep learning system delivered a sensitivity above 90% while maintaining a specificity below 5%, making automated analysis a viable option for clinicians. This framework for automatic analysis of EEGs can be applied in other classification tasks such as seizure detection or abnormal detection. There are many straightforward extensions of this system that can include more powerful deep learning networks such as Long Short-Term Memory Networks or Convolutional Neural Networks. This is the subject of our ongoing research.

This project is part of a long-term collaboration with the Department of Neurology at Temple University Hospital that has produced several valuable outputs including a large corpus (TUH-EEG), a subset of the corpus annotated for clinically relevant events (TUH-EEG-ESS), and technology to automatically interpret EEGs. In related work, we are also making the corpus searchable using multimodal queries that integrate metadata, information extracted from EEG reports and the signal event data described here (Obeid and Picone, 2016). The resulting system can be used to retrieve many different types of cohorts and will be a valuable tool for clinical work, research and teaching.

## Author Contributions

MG and AH: algorithm design, programmer; SL: data collection; IO: data collection, CO-PI; JP: data collection, algorithm design, PI.

### Conflict of Interest Statement

The authors declare that the research was conducted in the absence of any commercial or financial relationships that could be construed as a potential conflict of interest.
